# The effects of fructose and metabolic inhibition on hepatocellular carcinoma

**DOI:** 10.1038/s41598-020-73653-5

**Published:** 2020-10-07

**Authors:** Brittany Dewdney, Mohammed Alanazy, Rhys Gillman, Sarah Walker, Miriam Wankell, Liang Qiao, Jacob George, Alexandra Roberts, Lionel Hebbard

**Affiliations:** 1 Department of Molecular and Cell Biology, Centre for Molecular Therapeutics, Australian Institute of Tropical Health and Medicine, James Cook University, Townsville, QLD 4811 Australia; 2 Storr Liver Centre, Westmead Institute for Medical Research, Westmead Hospital and University of Sydney, Sydney, NSW 2145 Australia; 3 Gastroenterology and Hepatology Unit, The Canberra Hospital, Woden, ACT 2606 Australia

**Keywords:** Hepatocellular carcinoma, Cancer metabolism

## Abstract

Hepatocellular carcinoma is rapidly becoming one of the leading causes of cancer-related deaths, largely due to the increasing incidence of non-alcoholic fatty liver disease. This in part may be attributed to Westernised diets high in fructose sugar. While many studies have shown the effects of fructose on inducing metabolic-related liver diseases, little research has investigated the effects of fructose sugar on liver cancer metabolism. The present study aimed to examine the metabolic effects of fructose on hepatocellular carcinoma growth in vitro and in vivo*.* Fructose sugar was found to reduce cell growth in vitro, and caused alterations in the expression of enzymes involved in the serine-glycine synthesis and pentose phosphate pathways. These biosynthesis pathways are highly active in cancer cells and they utilise glycolytic by-products to produce energy and nucleotides for growth. Hence, the study further investigated the efficacy of two novel drugs that inhibit these pathways, namely NCT-503 and Physcion. The study is the first to show that the combination treatment of NCT-503 and Physcion substantially inhibited hepatocellular carcinoma growth in vitro and in vivo*.* The combination of fructose diet and metabolism-inhibiting drugs may provide a unique metabolic environment that warrants further investigation in targeting hepatocellular carcinoma.

## Introduction

Hepatocellular carcinoma (HCC) remains a universal health burden as it is the fourth leading cause of cancer-related deaths^1^. Although viral hepatitis and alcoholism account for a large majority of the HCC cases, there is an alarming increase in the incidence of HCC relating to non-alcoholic fatty liver disease (NAFLD) and non-alcoholic steatohepatitis (NASH)^2^. Prediction models suggest that the incidence of NAFLD/NASH-related HCC cases will increase 146% by 2030 in the United States^3^, and European countries show similar patterns of increasing prevalence of this disease^4^. This steep increase of HCC incidence will occur not just due to the aging population, but also as a result of the increasing prevalence of obesity and type 2 diabetes mellitus (DM).

Many have attributed the rise in obesity and DM to the overconsumption of sugar-rich foods associated with the Westernised diet. Particularly, the overconsumption of fructose sugar has been considered a major contributing factor to NAFLD development by causing increased liver de novo lipogenesis, inflammation and insulin resistance^5^. Several preclinical studies have suggested that fructose-rich diets can increase HCC incidence and tumour burden^6–9^. Furthermore, genetic analyses suggest that fructose induces metabolic changes to glycolysis that may fuel tumour growth^10,11^. Of unknown value are the contributions of fructose metabolism to the pentose phosphate pathway (PPP) and serine to glycine synthesis pathway (SGS), which are responsible for using glycolytic by-products to produce nucleotides and NADPH for cell growth and proliferation. The effects of fructose consumption on activating these pathways in HCC has yet to be fully evaluated.

Part of the burden of HCC can be attributed to the poor survival rate due to late state diagnoses, leaving very few treatment options. While there are approved drugs available for treating advanced stage disease such as Sorafenib, Regaforenib, and Lenvatinib, they are non-curative options and only extend life by 10–14 months^12^. With the growing incidence of HCC cases relating to obesity and NAFLD, and little advancements in the field of curative treatment, there is an urgent clinical need for novel therapeutic approaches in HCC therapy. Physcion is a naturally derived anti-cancer drug that inhibits the second enzymatic step in the PPP by blocking 6*-*phosphogluconate dehydrogenase (6PGD). Some recent studies have shown Physcion to be effective in reducing HCC growth in vitro^13,14^, yet no studies have investigated these effects in vivo*,* or in the combination of fructose supplementation. NCT-503 is a synthetic compound that inhibits the first enzymatic step of the SGS by blocking phosphoglycerate dehydrogenase (PHGDH). Currently only one study has investigated the effects of NCT-503 on HCC, demonstrating significant effects on reducing HCC growth in Sorafenib resistant tumours^15^.

In this study we investigated the effects of fructose on HCC cell growth in vitro and in vivo*.* Furthermore, we demonstrate for the first time the effects of PPP and SGS pathway inhibition using Physcion and NCT-503, respectively, in glucose and fructose grown HCC. Our results demonstrate that fructose induces metabolic changes in HCC growth, resulting in HCC cell death in vitro and varying drug response to metabolism inhibiting compounds in vivo. Moreover, the combination of both Physcion and NCT-503 caused a significant reduction of HCC tumour growth in vivo. In conclusion, we propose a novel role for fructose metabolism in HCC, and demonstrate that together with metabolism-inhibiting drugs, could represent a novel therapeutic strategy for treating NAFLD-related HCC.

## Results

### Fructose restricts HCC proliferation and induces metabolic changes to the PPP and SGS pathways

**Figure 1:**
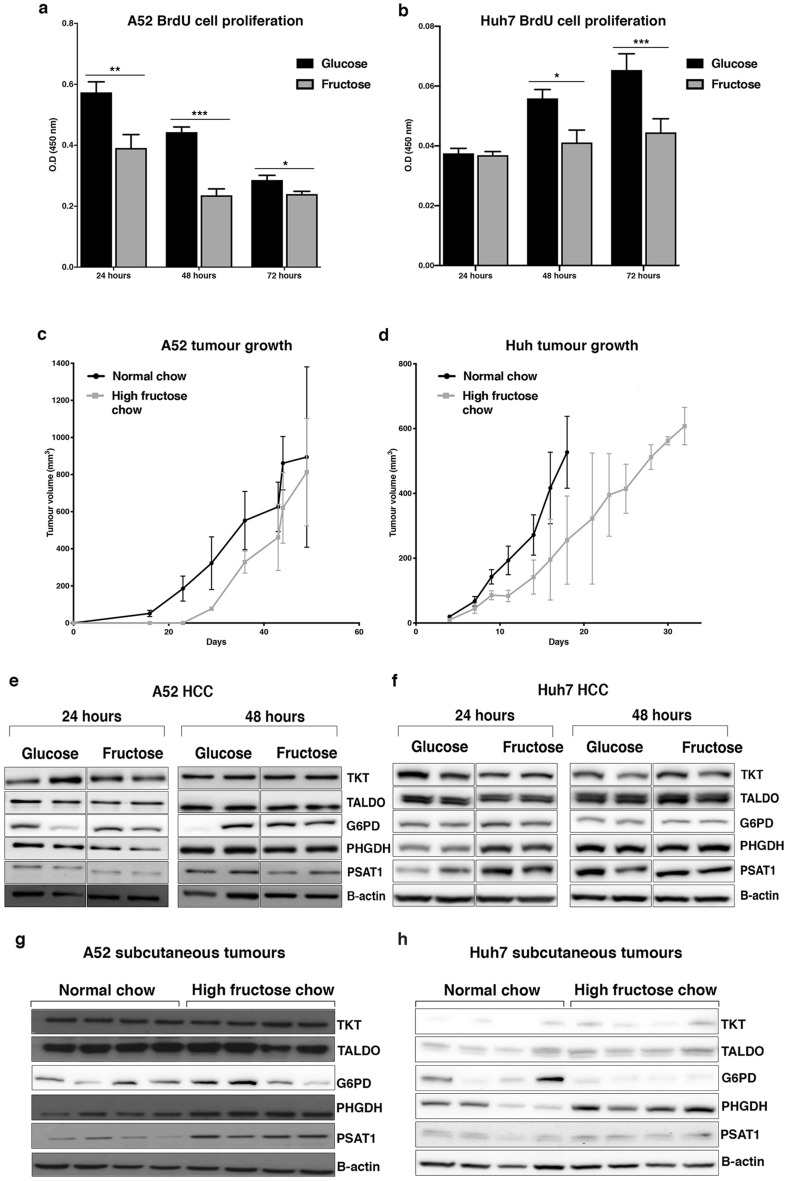
Evaluating A52 and Huh7 HCC growth and protein expression in glucose and fructose conditions. Cell proliferation via a BrdU assay was determined for (**a**) A52 cells, and (**b**) Huh7 cells grown in media containing 5 mM glucose or 5 mM fructose over 24, 48 and 72 h. Growth of (**c**) A52 and (**d**) Huh7 tumours was evaluated in mice fed a normal or high-fructose chow. Western blots of cell lysates for TKT, TALDO, PHGDH, G6PD, PSAT1 and β-actin from, (**e**) A52 cells, and (**f**) Huh7 cells growth in glucose or fructose containing media for 24 and 48 h, and tumour lysates from (**g**) A52, and (**h**) Huh7 (H) subcutaneous tumours in mice fed a normal chow or high fructose diet. Original blots and densitometry analysis for the in vitro protein analysis and in vivo protein analysis are shown in Supplementary Figures 1–8 (Supplementary File 1). Full length western blot images for TKT, TALDO, PHGDH, PSAT1 and β-actin could not be provided for (**e**) and (**g**). These images were provided from the thesis of M.A., and full-length original images could not be obtained. The original images in this figure and the Supplementary File are as presented in the thesis of M.A. *p < 0.05; **p < 0.01; ***p < 0.001; ****p < 0.0001. *G6PD* glucose-6-phosphate dehydrogenase, *PHGDH* phosphoglycerate dehydrogenase, *PSAT1* phosphoserine aminotransferase1, *TALDO* transaldolase, *TKT* transketolase.

Murine primary HCC cells, named A52, that we previously developed^16^, and human HCC Huh7 cells were used to study the effects of HCC growth in glucose and fructose supplemented media. Cells were cultured in glucose or fructose supplemented media and cell proliferation evaluated after 24, 48 and 72 h. Compared to glucose, HCC cells grown in fructose had significantly reduced cell proliferation (p < 0.05; Fig. 1a,b). Similarly, A52 and Huh7 subcutaneous tumour formation was respectively, slightly delayed or showed slower growth in mice fed a high fructose chow compared to those fed the normal chow (Fig. 1c,d). To explore the metabolic differences in glucose and fructose grown HCC, protein expression of enzymes involved in the PPP and SGS were evaluated from cell and tumour lysates. Fructose treated HCC cells showed little changes in expression of the SGS and PPP pathways (Fig. 1e,f and Supplementary Figures 2 and 4). However, through dietary intake fructose increased expression of the SGS rate limiting enzyme, PHGDH, as well as the second SGS enzyme PSAT1 (phosphoserine aminotransferase1), in A52 and Huh7 tumours (Fig. 1g,h and Supplementary Figures 6 and 8)*.* Enzymes of the non-oxidative branch of the PPP, namely transketeloase (TKT) and transaldolase (TALDO) remained unchanged in vivo. Interestingly, the expression of glucose-6-phosphate dehydrogenase (G6PD), the rate limiting enzyme of the oxidative branch of the PPP, was upregulated in fructose A52 tumours (Fig. 1g and Supplementary Figure 6)*,* while downregulated in the Huh7 fructose group (Fig. 1h and Supplementary Figure 8).

### The PPP is upregulated in HCC and is associated with poor patient survival

**Figure 2:**
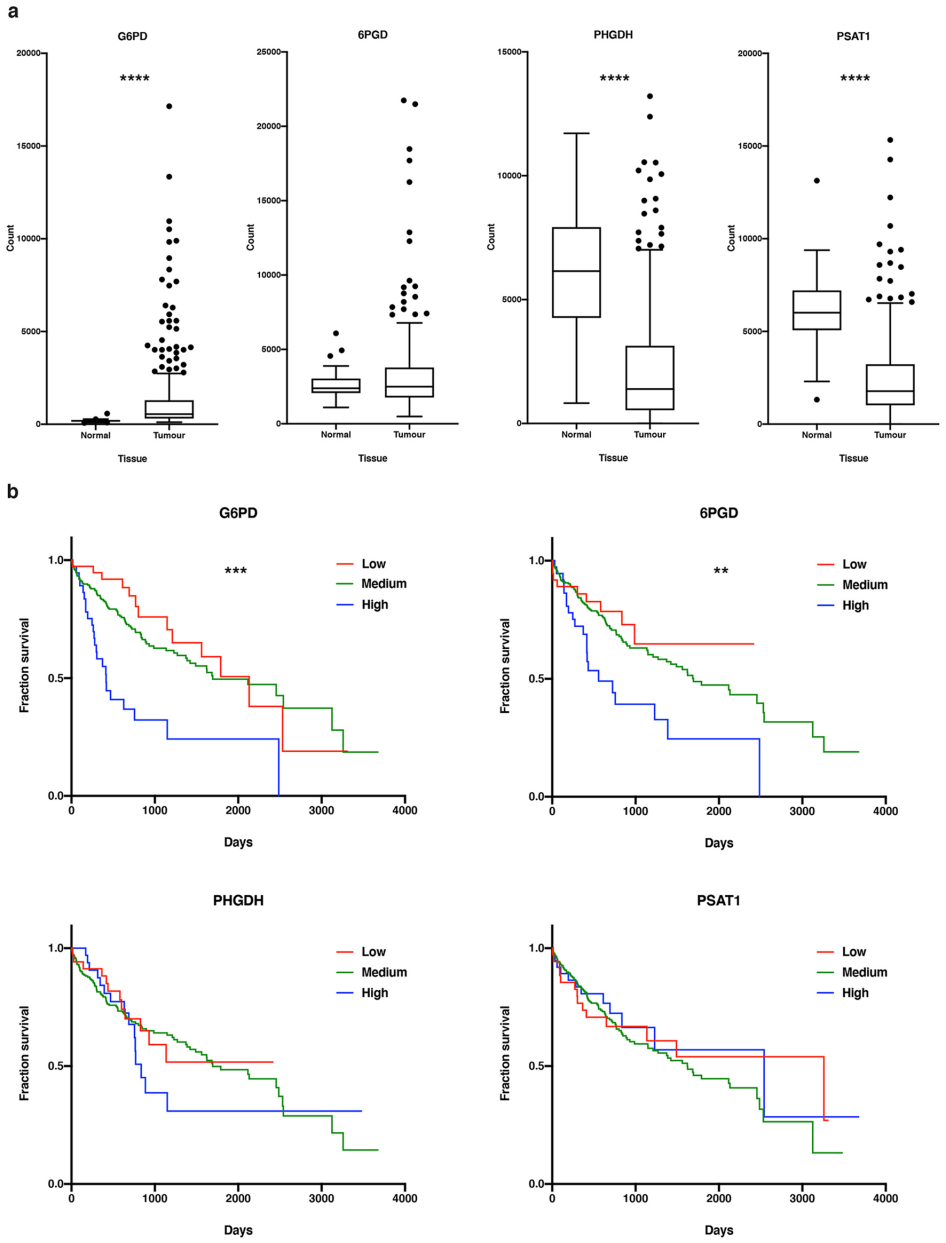
Evaluation of the PPP and SGS in human HCC. (**a**) Gene expression of G6PD, 6PGD, PHGDH, and PSAT1 was determined in HCC tumour tissue (n = 371) and normal liver tissue (n = 50). Boxplots show median represented by the horizontal line and the box boundaries represent the first and third quartile range. The whiskers are derived from the Tukey method. (**b**) Patients were sorted into percentiles of low, medium, and high expression of G6PD (low n = 38, medium n = 293, high n = 39), 6PGD (low n = 38, medium n = 292, high n = 40), PHGDH (low n = 38, medium n = 292, high n = 40), and PSAT1 (low n = 38, medium n = 191, high n = 40), and graphs represents overall patient survival in low, medium, and high expression groups. *p < 0.05; **p < 0.01; ***p < 0.001; ****p < 0.0001. *6PGD* 6-phosphogluconate dehydrogenase, *G6PD* glucose-6-phosphate dehydrogenase, *PHGDH* phosphoglycerate dehydrogenase, *PSAT1* phosphoserine aminotransferase 1.

To determine the association of the PPP and SGS pathways in clinical HCC, we analysed PHGDH, PSAT1 and G6PD RNA expression in HCC tumour and non-tumour liver tissue from the Cancer Genome Atlas (TCGA) liver hepatocellular carcinoma (LIHC) database. We also considered the second enzyme of the PPP pathway, 6PGD (6*-*phosphogluconate dehydrogenase), as it represents a novel HCC metabolic target. Between the two groups, tumour tissue had significantly upregulated G6PD (p < 0.0001), unaltered 6PGD, and PHGDH and PSAT1 were significantly downregulated (p < 0.0001), versus the non-tumour tissue (Fig. 2a). Considering survival, patients with high expression of either G6PD or 6PGD have significantly decreased overall survival compared to those with low or medium expression (p < 0.01). With regards to PHGDH or PSAT1, there was no difference in overall survival for patients with low, medium, or high expression (Fig. 2b).

### Fructose-grown HCC is inhibited by NCT-503 and Physcion in vitro

**Figure 3:**
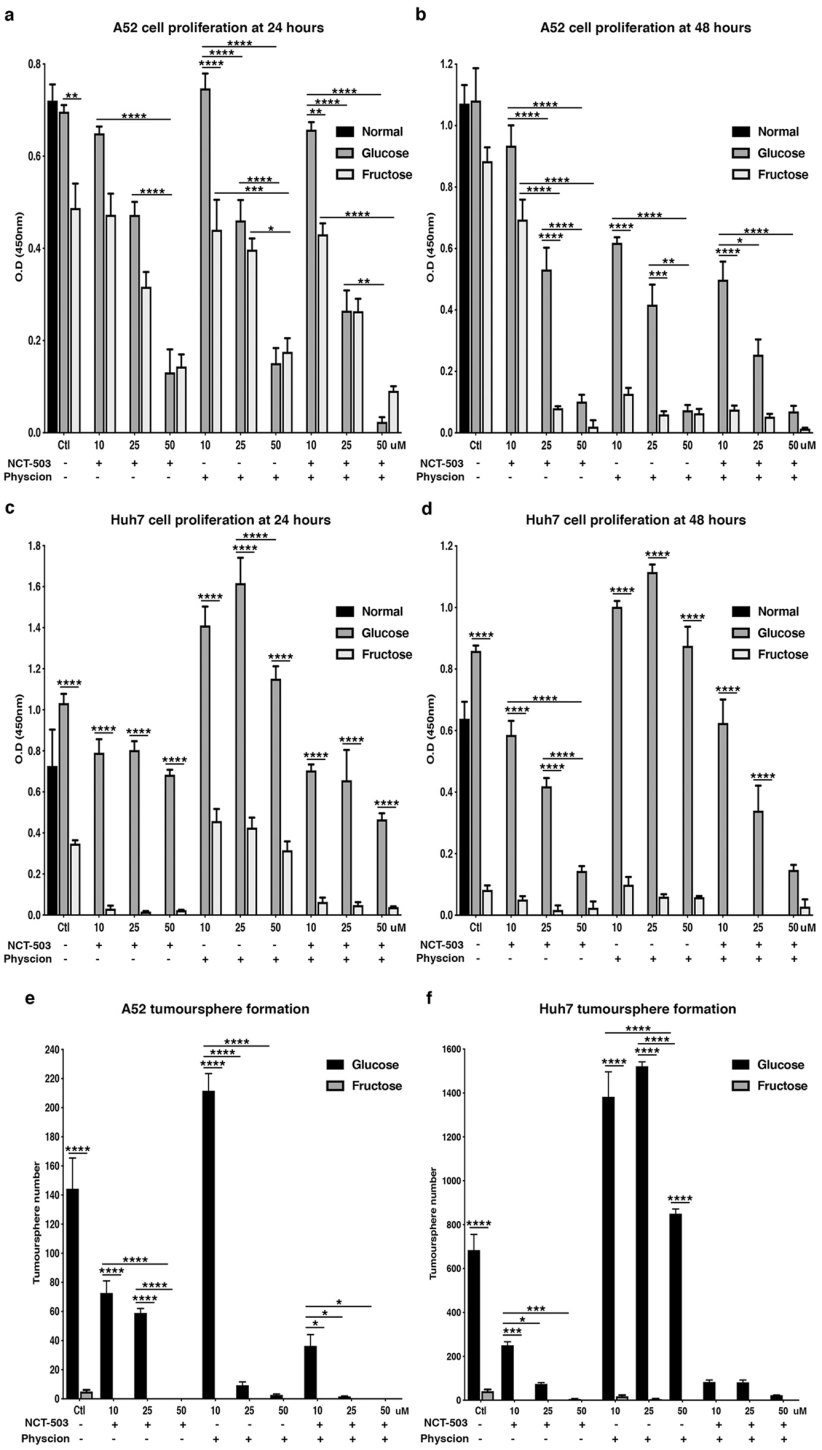
A52 and Huh7 cell proliferation with NCT-503 and Physcion. Cell proliferation was determined using the BrdU assay in cells grown in either normal media, media containing 5 mM glucose or 5 mM fructose, with or without 10, 25, or 50 μM of NCT-503 and/or Physcion. Graphs represent the average optical density at 450 nm (n = 4) for (**a**) A52 cells at 24 h; (**b**) A52 cells at 48 h; (**c**) Huh7 cells at 24 h; and (**d**) Huh7 cells at 48 h, after drug treatments. Tumoursphere number represents, (**e**) A52 cells, and (**f**) Huh7 cells grown in media containing 5 mM glucose or 5 mM fructose with or without 10, 25, or 50 μM of NCT-503 and/or Physcion. Data represents average sphere number per treatment group (n = 3). *p < 0.05; **p < 0.01; ***p < 0.001; ****p < 0.0001. *Ctl* control.

As our in vivo tumour data and bioinformatic analyses suggested relationships between the PPP and SGS on HCC growth, we inhibited the PPP and SGS with Physcion and/or NCT-503, respectively, and determined A52 and Huh7 cell proliferation under glucose and fructose conditions. Consistent with previous results, fructose grown HCC cells had reduced growth compared to normal and glucose grown cells (Fig. 3). Importantly, after 24 and 48 h, both A52 and Huh7 cells treated with fructose and either NCT-503, Physcion, or in combination significantly reduced cell proliferation compared to glucose (p < 0.0001). A52 cells treated with glucose and increasing concentrations of NCT-503 and Physcion (25 and 50 μM) had significantly reduced growth compared to control at 24 and 48 h (p < 0.01; Fig. 3a,b). Unexpectedly, Huh7 cells had increased proliferation in the presence of Physcion and glucose, while combination treatment diminished growth similarly to that seen with NCT-503 alone (Fig. 3c,d). In contrast to A52 cells, Huh7 growth was significantly reduced after 48 h of exposure to low concentrations of NCT-503 (p < 0.001; Fig. 3b,d). This data is supported by tumoursphere assays of A52 and Huh7 cells. Fructose alone dramatically reduced sphere number in A52 and Huh7 cells (Fig. 3e,f). Similarly, Huh7 tumoursphere number increased in the presence of Physcion and glucose, while NCT-503 significantly reduced sphere formation (p < 0.0001; Fig. 3f). Collectively, these results suggest A52 cells are more sensitive to PPP inhibition, while in contrast Huh7 cells are more affected by SGS inhibition, and the effect of metabolic pathway inhibition in conjunction with fructose feeding is detrimental for HCC cell growth in vitro.

### Fructose induces apoptosis in HCC in vitro

**Figure 4:**
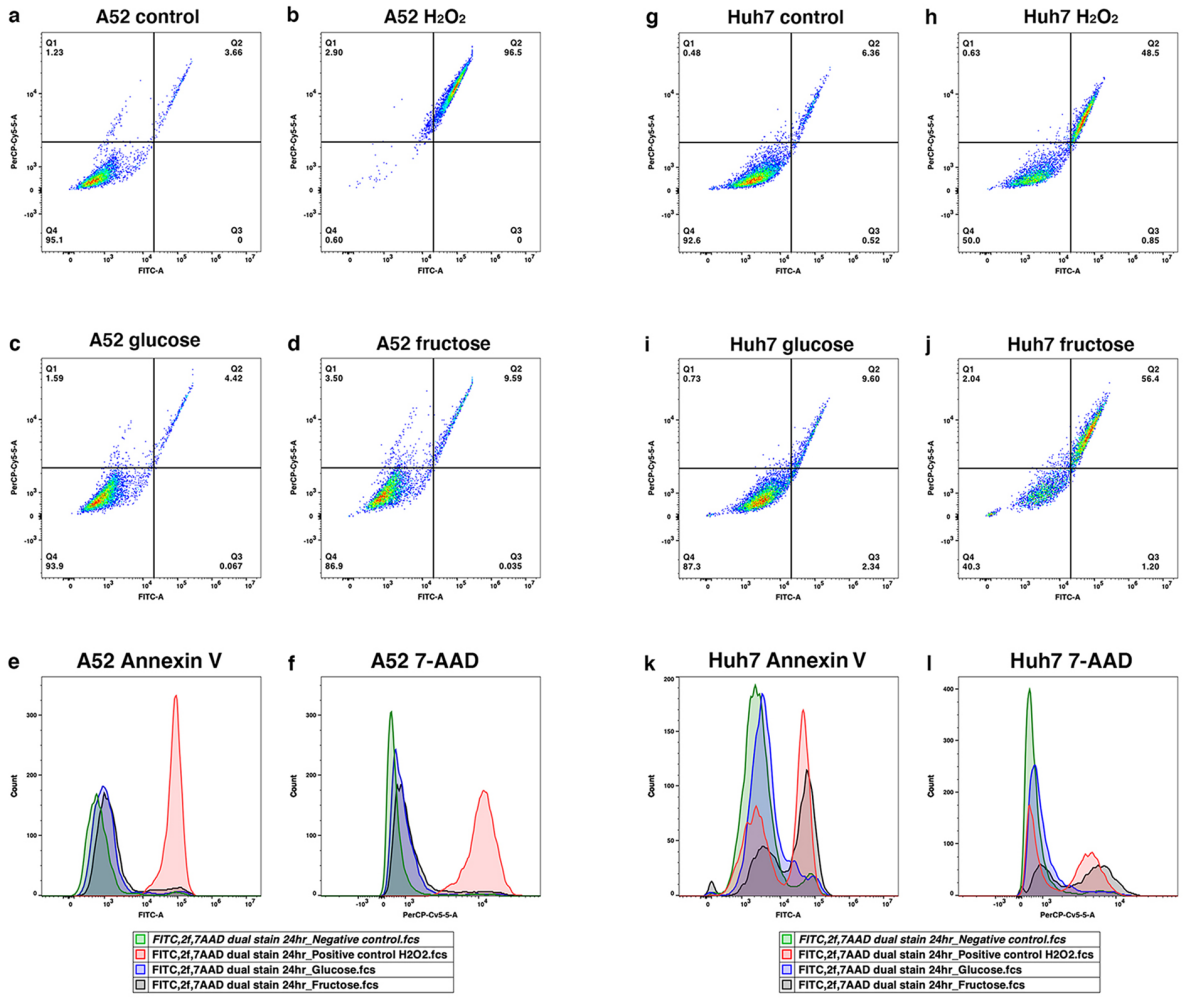
A52 and Huh7 FACS analysis of apoptosis in glucose and fructose conditions. A52 and Huh7 cells were grown in either normal media, media containing 5 mM glucose, or 5 mM fructose for 48 h, and stained with 7-AAD and FITC-Annexin V to analyse apoptotic cell populations. For a positive control, cells were incubated with 1 mM H_2_O_2_ for 2 h prior to FACS to induce apoptosis. Data represent the stained cell population percentages of 10,000 events for normal media (**a**,**g**), positive control (**b**,**h**), glucose media (**c**,**i**), and fructose media (**d**,**j**) for A52 and Huh7, respectively. Apoptosis positive cells are visible on the FITC axis in quadrants Q2 and Q3. Cells that are dead or in late stages of apoptosis with increased 7-AAD DNA binding are shown on the PerCP-Cy5-5 axis in quadrants Q1 and Q2. Histogram plots show cell counts for Annexin V and 7-AAD for A52 (**e**,**f**) and Huh7 (**k**,**l**), respectively.

To investigate the mechanism by which fructose reduced cell proliferation in vitro, A52 and Huh7 cells grown in glucose or fructose were stained with Annexin V and 7-AAD, and analysed with flow cytometry. Control apoptosis was induced with H_2_O_2_ (Fig. 4b,h). Compared to control cells grown in normal media, glucose treated cells had similar cell viability for A52 (Fig. 4a,c) and Huh7 cells (Fig. 4g,i). In contrast, fructose promoted a 2.6-fold increase in A52 apoptotic cells (Fig. 4d), and an 8.8-fold increase in Huh7 apoptotic cells (Fig. 4j). Histogram plots show a strong second peak shift for FITC and 7-AAD in fructose treated Huh7 cells (Fig. 4k,l), and only small peaks for A52 cells (Fig. 4i,j). This parallels the cell proliferation data, showing Huh7′s have markedly reduced cell proliferation after 48 h with fructose media (Fig. 3d), whereas A52 cells only had a slight reduction in growth in fructose (Fig. 3b).

### NCT-503 and Physcion inhibit HCC tumour growth in vivo

**Figure 5:**
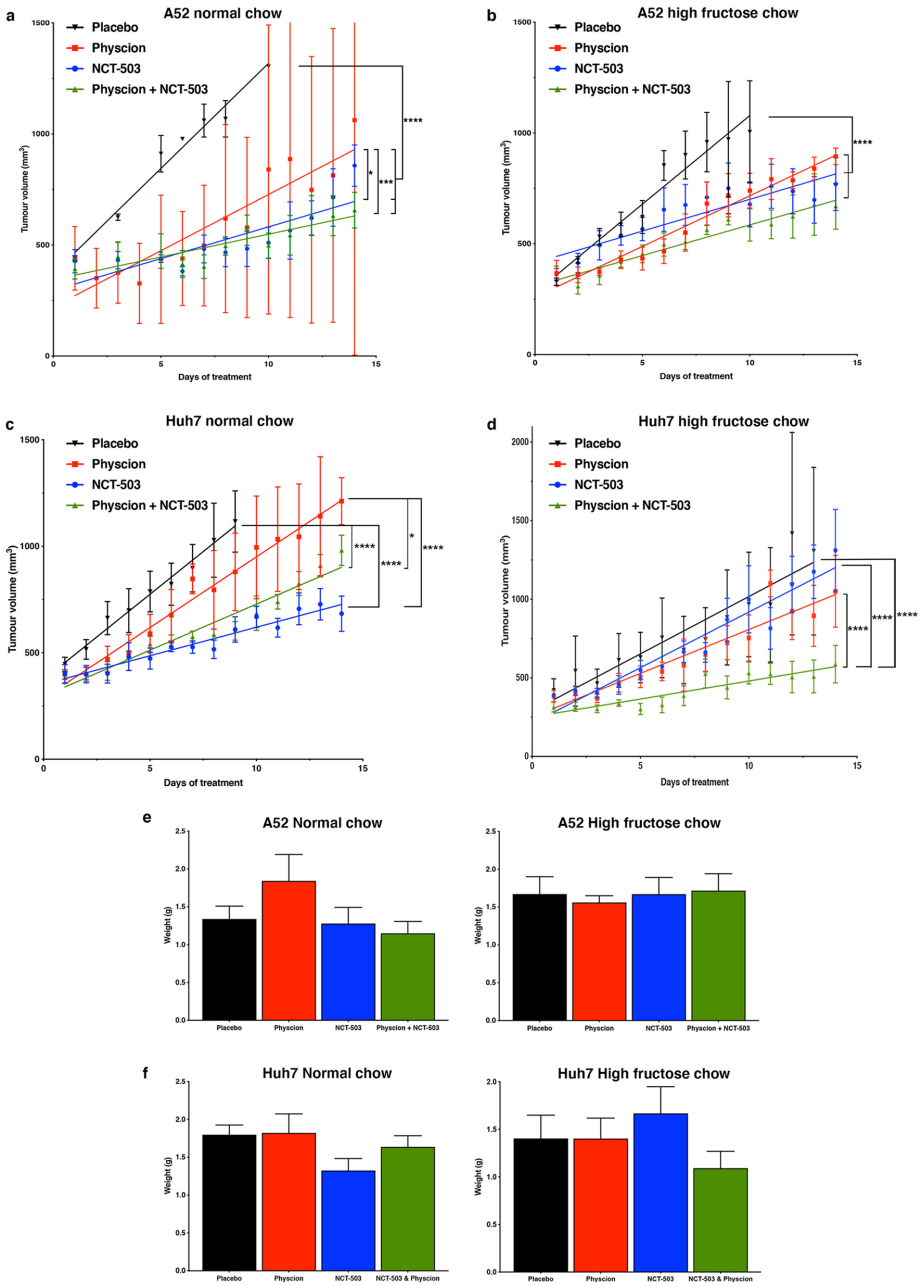
Effect of NCT-503 and Physcion on glucose and fructose fed HCC tumour growth. Tumour growth curves of mice with (**a**) normal chow and A52 HCC cells, (**b**) high fructose chow and A52 HCC cells, (**c**) normal chow and Huh7 HCC cells, and (**d**) high fructose chow and Huh7 HCC cells, and treatment with placebo (black inverted triangles),40 mg/kg NCT-503 (blue circles), 20 mg/kg Physcion (red squares), and 40 mg/kg NCT-503 + 20 mg/kg Physcion (green upright triangles). Data represent the average daily tumour volume over a 14-day treatment period. (**e**) A52 and (**f**) Huh7 represent the final average tumour weight for groups fed a normal or high fructose diet. Groups: n = 6–12 tumours/group; *p < 0.05; **p < 0.01; ***p < 0.001; ****p < 0.0001.

Given these data and to give relevance to patients harbouring HCC, we initiated therapy of established 500 mm^3^ A52 and Huh7 subcutaneous tumours in mice fed either a normal or high fructose chow, and treated with either Physcion, or NCT-503, or in combination. After two weeks of treatment, A52 tumours had significantly reduced tumour growth in all treatment groups compared to the placebo group, in both the normal chow and fructose chow-fed mice (p < 0.0001; Fig. 5a,b). Moreover, the combination treatment and NCT-503 alone had a greater affect in reducing A52 tumour growth compared to Physcion alone (Fig. 5a). After the 14-day treatment there was no apparent differences in the rate of tumour growth between the A52 normal chow and fructose groups (Fig. 5a,b), and there was no significant difference in final tumour weights between the A52 treatment groups (Fig. 5e).

Similar to the in vitro results, in the normal chow fed mice Huh7 tumour growth was significantly inhibited by NCT-503 alone and the combination treatment group, while the Physcion treatment group had similar growth to placebo (p < 0.0001; Fig. 5c). In mice fed a high fructose chow, only the combination treatment significantly reduced tumour growth compared to the placebo group (p < 0.0001), and NCT-503 had little effect on reducing tumour growth (Fig. 5d). Similar to the A52 tumours, no reduction in the rate of Huh7 tumour growth was observed in fructose-fed mice compared to the normal chow. In comparing final tumour weights, there were non-significant reductions for the normal chow NCT-503 and high fructose combinatorial groups, and no noted differences in the other groups (Fig. 5e,f). This is likely due to the variable and only moderate effects of the drugs on delaying tumour growth, particularly in the A52 model (Fig. 5a,b).

**Figure 6:**
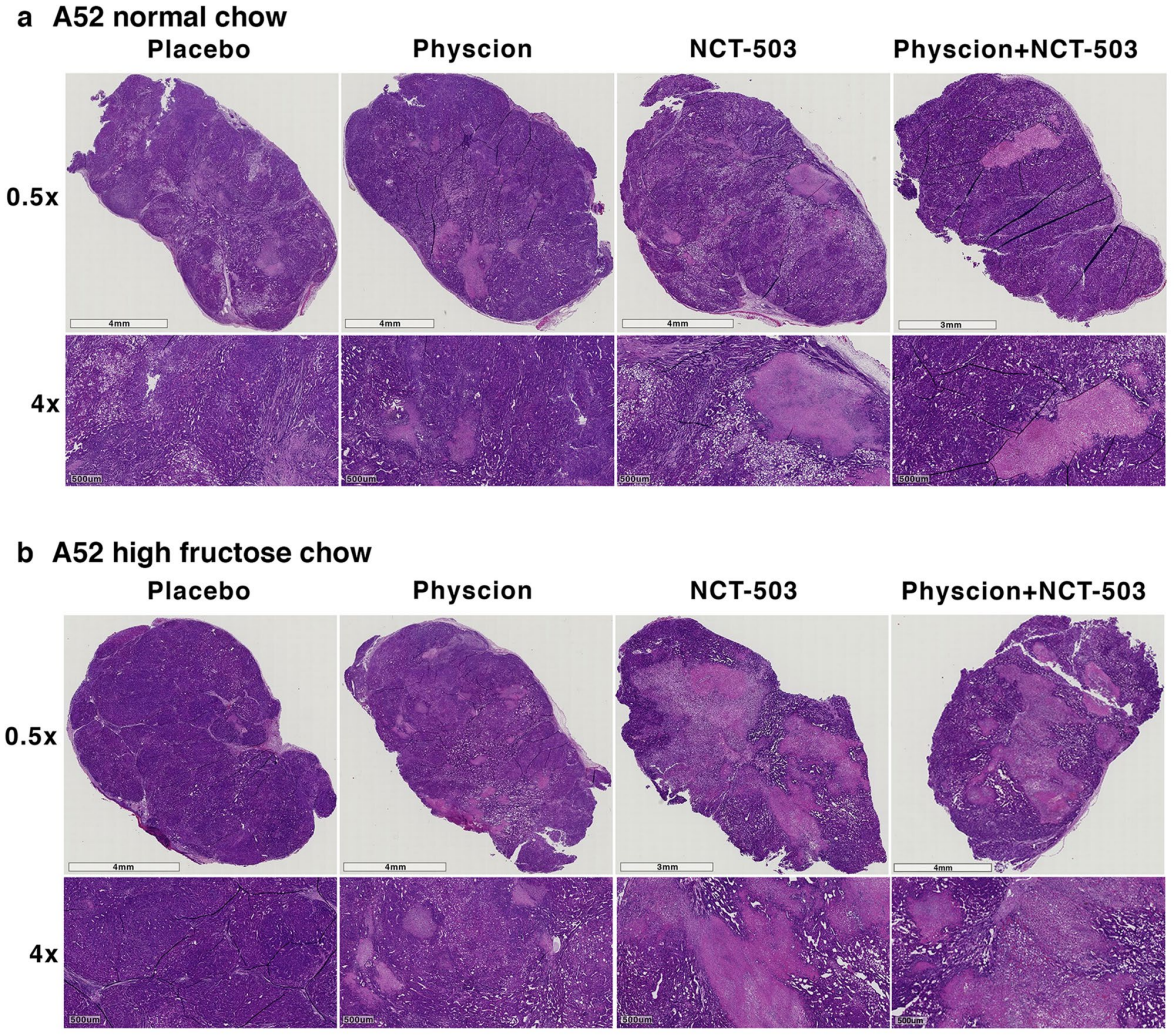
A52 tumour histology treated with NCT-503 and Physcion. Haematoxylin and eosin images representing A52 pathology from each designated treatment group.

**Figure 7:**
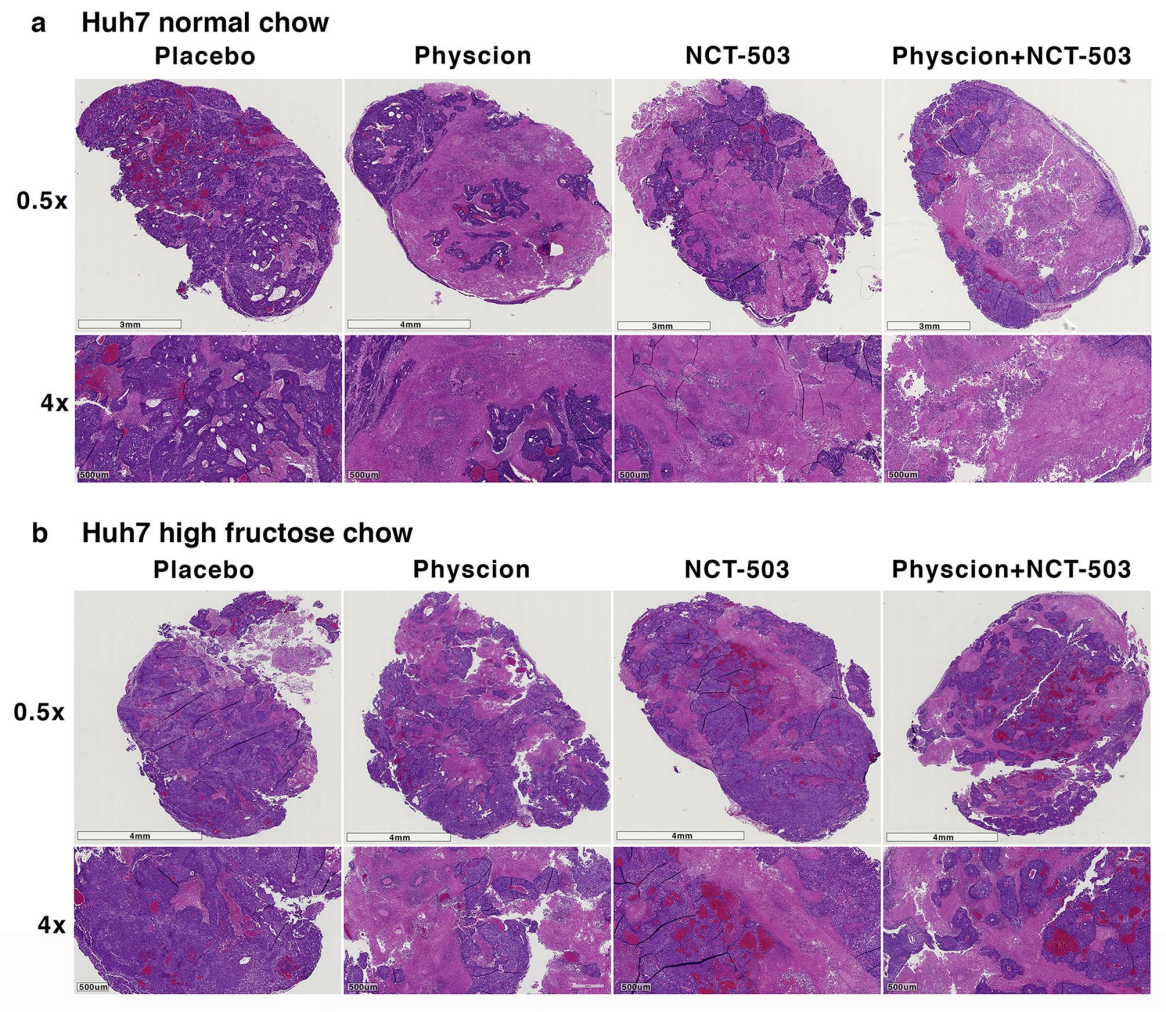
Huh7 tumour histology treated with NCT-503 and Physcion. Haematoxylin and eosin images representing Huh7 pathology from each designated treatment group.

To gauge changes in tumour morphology A52 and Huh7 tumours were fixed and stained with haematoxylin and eosin. Regardless of diet or treatment, A52 tumours showed little change in tumour morphology (Fig. 6). In normal chow Huh7 tumours, there was a decreasing trend of haematoxylin staining in the drug-treated tumours (Fig. 7), however overall this was non-significant. Haematoxylin quantification showed there were no significant changes in haematoxylin ratios in the A52 or Huh7 tumours (Supplementary Figure 9).

## Discussion

To our knowledge, this is the first study examining the effects of fructose on HCC proliferation and growth in in vitro and in vivo models. We demonstrate that fructose significantly restricts HCC cell proliferation in vitro and causes metabolic adaptation in terms of protein expression of the PPP and SGS pathways. Through bioinformatic analyses of human data sets we find that increased G6PD and 6PGD gene expression associates with reduced survival. Hence, to address the activity of both the PPP and SGS pathways in HCC, we treated two subcutaneous tumour models for 2 weeks with a combinatorial strategy of Physcion and NCT-503. Together these two drugs can promote a significant reduction in HCC tumour growth.

The role of fructose in HCC development remains an unclear and controversial topic. While it is well established that excess fructose can lead to fatty liver, insulin resistance and liver inflammation, it is unknown how fructose metabolism affects HCC development. Our work demonstrates that under in vitro conditions, fructose sugar causes a significant retardation in murine and human HCC cell proliferation and tumoursphere formation. Fructose metabolism in hepatocytes is largely ATP-dependent and the breakdown of fructose into fructose-1-phosphate causes rapid depletion of ATP in the cells^17^. In addition, the depletion of cellular ATP indirectly causes the accumulation of uric acid, which stimulates the production of reactive oxygen species (ROS) and induces mitochondrial and endoplasmic reticulum oxidative stress^18^. Therefore, the most likely cause for the dramatic reduction in cell proliferation and sphere number in fructose-fed HCC in vitro is due to increased cell death as a result of insufficient energy and ROS accumulation. In contrast, in the presence of high fructose feeding our in vivo models did not exhibit considerable changes in tumour growth. This could be a product of our chosen model where organs, such as the liver and muscle, are more involved in metabolizing fructose rather than the subcutaneous tumour, leading to a reduction in the expected deleterious effects of fructose, and that the subcutaneous tumour has developed an alternative pathway to support growth. It is also likely that the subcutaneous HCC tumour may be utilising adipose or muscle-derived compounds from surrounding tissue, such as lactate, alanine or pyruvate, to sustain growth during high-fructose feeding^19^. This phenomenon could also partly explain the moderate effects the metabolism-inhibiting drugs had in vivo compared to in vitro. We briefly investigated this by examining cell proliferation of A52 and Huh7 cells exposed to NCT-503 and Physcion with or without the addition of amino acid supplementation. Our results show that the presence of amino acids in the cell media partly attenuated the inhibitory effects of NCT-503 and Physcion, in both glucose and fructose containing media (Supplementary Figure 10). These observations also support the concept that HCC metabolism is dynamic and adaptable to the tumour microenvironment.

Similarly, differential expression of metabolic enzymes has been noted between the primary tumour and metastases. By example, a recent study showed that liver metastases derived from colorectal cancer had increased aldolase B expression, an enzyme involved in fructose metabolism. Moreover, the authors demonstrated that the metastases had reprogrammed metabolism to activate the fructose metabolic pathway, and a high fructose diet increased colorectal liver metastasis^20^. Conversely, another study showed reduced aldolase B expression and fructose metabolism in primary HCC, and that overexpression of aldolase B reduced HCC metastasis, and was associated with improved patient survival^21^. Additionally, studies have implicated the down-regulation of the fructose metabolic enzymes ketohexokinase-C and fructose-1,6-biphosphatase in promoting glycolysis and cancer growth, and suggest fructose metabolism is unfavourable in HCC^22–24^. Here, in the context of our models we show that fructose causes a metabolic shift in the SGS and PPP pathways, but had no effect on HCC growth in vivo*.*

Our work has shown that a novel inhibitor of the SGS pathway, NCT-503, has the potential to inhibit HCC in vitro and in vivo. Only a few studies have investigated the use of NCT-503 in cancer growth inhibition. Nevertheless, the original work describing the action of NCT-503 on SGS inhibition, illustrated that NCT-503 was only effective in PHGDH-dependent cell lines, with little or no impact on PHGDH-independent cell lines in vitro or in vivo^25^. This may in part explain the moderate effects of NCT-503 on HCC growth, and the variation we observed between our murine and human HCC models. Furthermore, this study showed that NCT-503 only affected glucose-derived production of serine, thereby inhibiting nucleotide synthesis originating from glucose^25^. Additionally, when exogenous sources of serine were supplied, NCT-503 had a limited effect on inhibiting the production of nucleotides^25^. This in part supports our results that demonstrate the strong impairment of HCC proliferation after treatment with NCT-503 in vitro with only moderate effects in vivo. Given glucose is the major source of fuel in vitro, HCC cells are more sensitive to SGS inhibition where glucose is the endogenous carbon source of glycolysis. However, considering our subcutaneous HCC models, it is likely that other exogenous sources of serine are available. This could in part explain why NCT-503 treatment was modestly effective in normal chow conditions in A52 and Huh7 tumours, and less effective when fed fructose. Similar results have been seen in another study, where NCT-503 treatment of HCC had only moderate effects in reducing subcutaneous tumour growth^15^. This result could be expected, as our bioinformatics data suggests that even though the SGS pathway is downregulated in HCC patients, the relative expression appears to be unrelated to patient survival.

Recent studies have investigated the efficacy of Physcion in reducing HCC growth, and they contradict our current work. Studies on the action of Physcion with Huh7 cells, show that it reduces cell proliferation and subcutaneous tumour growth^13,26,27^. Here, we demonstrate that Physcion promoted Huh7 cell growth at low concentrations in vitro*,* while having minimal effects on reducing tumour growth. Although we observed increases in haematoxylin staining in the centre of Physcion-treated tumours, to suggest cell death, the best explanation for our in vivo observations is that we evaluated tumour growth from 500 mm^3^ to better mimic the clinical situation. It is unclear why we observed an increase in HCC growth with Physcion in vitro*,* as this was an unexpected result and did not coincide with our protein expression data. Nonetheless, our bioinformatics data supports the concept that HCC patients have increased PPP activity and that high expression of the PPP enzymes G6PD and 6PGD are associated with poor patient survival. Thus, targeting this pathway with Physcion or other inhibitors warrants further study, and could perhaps be more effectively used in earlier tumour development.

The limitation of this study is the moderate summative effects of NCT-503 and Physcion on HCC tumour growth. Although there was a substantial change in tumour growth rates, the tumour morphology is not suggestive of extensive cell death. Therefore, the use of these drugs in HCC patients may only be of value in early stage HCC, as an adjuvant therapy or as a treatment to slow tumour growth in preparation for resection. For example, a study showed that Sorafenib resistant HCC have increased SGS activity and PHGDH expression, and subsequent combinatorial treatment with Sorafenib and NCT-503 may overcome HCC drug resistance^15^. Similarly, another study showed the combination of Physcion and Sorafenib can inhibit HCC growth better than Sorafenib alone^28^. In our study, we did not begin drug treatment until tumours were half the maximal size in order to mimic advanced HCC stages observed in the clinic. Moreover, given the apparent activity of these inhibitors using smaller subcutaneous tumours, perhaps our experimental approach of using more advanced HCCs should be considered as the status quo to more carefully evaluate new HCC targets. Taken together, these drugs may not induce regression in advanced tumours, but their combination may be more effective in early stage HCCs.

In conclusion, this study is the first to utilise a high fructose diet as means to manipulate the metabolic environment of HCC tumour growth. While many studies suggest high fructose promotes HCC development, our work indicates that fructose sugar may not entirely support HCC growth, and rather promotes metabolic adaptation in HCC tumours to utilise other carbon sources for energy. In this manner, we have shown that the activity of biosynthesis pathways involved in cancer cell growth, namely the SGS and PPP pathways, may be regulated through nutrition in HCC. Furthermore, manipulating the activity of the SGS and PPP allows for a unique metabolic environment that may be targeted with two novel inhibitors, NCT-503 and Physcion. The combination of NCT-503 and Physcion may be a novel treatment option for reducing HCC tumour growth, and warrants further investigation for their efficacy in a clinical setting.

## Methods

### Bioinformatics

RNASeq and patient outcome data was retrieved from the Broad GDAC Firehose via the Firebrowse data portal for the Cancer Genome Atlas (TCGA) liver hepatocellular carcinoma (LIHC) cohort (https://gdac.broadinstitute.org/); and use of the TCGA dataset was approved by the NIH as part of our project entitled #21012: "Investigating Novel Genes in HCC”. RNASeq data was normalised and differential expression of genes of interest between tumour and normal samples was evaluated using the R/Bioconductor package ‘DESeq2’, with significant differential expression defined by an adjusted p value < 0.05. Based on gene expression in tumour tissue, patients were separated into cohorts with ‘High’, ‘Medium’, or ‘Low’ expression of each gene of interest. Using patient outcome data, patient median survival was calculated and plotted using the R/Survival package, and significant difference in survival between cohorts was calculated using the log-rank test.

### Cell Culture

Huh7 HCC cells were cultured in DMEM media supplemented with 10% heat-inactivated FBS and 1% (v/v) Penicillin–Streptomycin (Gibco). A52 HCC cells were used for a murine model of HCC as published by us^16^ and cultured in DMEM supplemented with 20% heat-inactivated FBS, 20 μg/L human epidermal growth factor (hEGF; Sigma Aldrich), 0.01 g/L insulin (Gibco), 0.01 g/L hydrocortisone, 1 mM phenobarbital, and 1% (v/v) Penicillin–Streptomycin. Cells were maintained at 37 °C in 5% CO_2_.

### Western blot

Cells and tumour tissue were lysed in RIPA buffer (50 mM Tris, pH 7.5, 150 mM NaCl, 1% NP40, 0.5% sodium deoxycholate, 0.1% SDS, 2 mM sodium orthovanadate, 50 mM NaFl, 1 mM sodium molybdate, 40 mM β-glycero-phosphate, 1 mM PMSF and 1/100 protease inhibitor cocktail) and protein concentration of lysates was determined using DC Protein Assay Kit (Bio-Rad). A total of 25 μg of protein was subjected to gel electrophoresis on a 10% SDS-PAGE gel and transferred to a PVDF membrane for one hour at 100 V. Membranes were washed in TBST (Tris-buffered saline with 0.2% Tween 20) and blocked with 5% skim-milk/TBST. Membranes were incubated at 4 °C overnight with the following primary antibodies: G6PD (12263, Cell Signaling), PSAT1 (ab96136, Abcam), PHGDH (ab13428, Abcam), transketolase (8616, Cell Signalling), transaldolase (PA5-27614, ThermoFisher) and β-actin (A2228, Sigma Aldrich), Membranes were washed in TBST and incubated with their respective HRP-conjugated secondary antibodies (Sigma) in 5% skim-milk/TBST, washed with TBST and visualised using SuperSignal West Pico PLUS Chemiluminescent Substrate kit (34577, ThermoFisher). Original blot images can be found in Supplementary File 1. Densitometry analysis was performed using ImageJ and normalised to β-actin expression to determine the adjusted density value for each protein.

### In vitro effect of fructose, NCT-503, and Physcion on HCC cell proliferation

Huh7 and A52 cells were seeded at 1.0 × 10^4^ cells and 2.0 × 10^3^ cells per well, respectively, in 96-well plates. After 24-h, the media was replaced with fresh media containing either standard media, glucose-free DMEM media (Gibco) supplemented with 10% heat-inactivated dialysed FBS, and 5 mM glucose or 5 mM fructose. For drug experiments, the media was supplemented with 10, 25, or 50 μM of NCT-503 (HY-101966, MedChemExpress) and/or Physcion (M4323, Abmole). Amino acid media supplement was a 1/100 dilution of MEM non-essential amino acid solution (M7145, Sigma Aldrich). All treatment groups were conducted in quadruplicate, and cell proliferation was determined after 24-h and 48-h with a BrdU Colorimetric assay (Roche), according to the manufacturer’s protocol.

### In vitro effect of NCT-503 and Physcion on HCC tumoursphere formation

Huh7 and A52 cells were cultured in sphere assay media consisting of 2% B27 Supplement (Gibco), 20 ng/mL hEGF, 10 ng/mL basic fibroblast growth factor (bFGF; Gibco), 2 μg/mL heparin (Sigma Aldrich), 5 μg/mL insulin, 0.5 μg/mL hydrocortisone, and 1% (v/v) Penicillin–Streptomycin. Huh7 and A52 cells were seeded at 3.0 × 10^4^ cells per well in 6-well low attachment plates (Sigma Aldrich) and incubated at 37 °C in 5% CO_2_ for seven days. Treatment groups included sphere formation in media containing either 5 mM glucose or fructose alone, or glucose or fructose-supplemented media containing NCT-503 and/or Physcion. After 7 days, tumourspheres were counted.

### FACS analysis

Huh7 and A52 cells were cultured in normal media (negative control), glucose media, or fructose media, as described above, for 48 h. For a positive control, fresh media containing 1 mM H_2_O_2_ was supplied to A52 and Huh7 cells 2 h prior to experimentation to induce apoptosis. For apoptotic cell analysis, cells were harvested with Accutase and stained with FITC-Annexin V (640906, BioLegend) and 7-AAD (7-aminoactinomycin D; 420404, BioLegend). Flow cytometry was performed using a BD LSRFortessa and BD FACSDIVA software (Version 6.1.3, BD Sciences). The FITC channel and PerCP-Cy5-5 channel were used for Annexin V and 7-AAD emission wavelengths, respectively. A total of 10,000 events were recorded for each group and FlowJo version 10.6.2 was used for data analysis and graphing. Quadrant placement (FITC-A versus PerCP-Cy5-5) was based on unstained and single stained cells from the negative and positive control treatment groups, and represent Q1 (non-specific cell death), Q2 (late stage apoptosis), Q3 (early stage apoptosis) and Q4 (viable cells).

### In vivo effect of NCT-503 and Physcion in glucose and fructose-fed HCC tumours

All animal experimental protocols were approved by the James Cook University Animal Welfare and Ethics committee (A2238). All experiments were performed in accordance with relevant guidelines and regulations. Male C57BL/6 mice or male BALB/cFox1nu mice aged 6–12 weeks were given ad libitum access to food and water. Huh7 and A52 HCC tumour growth was evaluated in male BALB/cFox1nu and male C57BL/6 mice, respectively. Mice were fed isocaloric diets of a normal chow (AIN93G, Specialty Feeds; protein 19.4%, fats 7%, carbohydrate 56.8%, crude fibre 7% and acid detergent fibre 7%) or a 60% fructose chow (SF03-018, Specialty Feeds; where fructose replaced sucrose, dextrinised starch and starch) for three weeks prior to HCC cells (2.5 × 10^6^ cells in 200 μL PBS/ECM gel; Sigma Aldrich) being bilaterally and subcutaneously injected into the rear flanks of the mice (n = 6–12 each group). For drug experiments, tumours were allowed to grow to a half-maximum size of 500 mm^3^ to mimic tumours observed in the clinic prior to drug treatment. Mice were randomly allocated into the following treatment groups: (i) placebo (vehicle only), (ii) 40 mg/kg NCT-503 (dissolved in 60% of 30% 2-hydroxypropyl-β-cyclodextrin, 30% PEG-300, and 10% ethanol), (iii) 20 mg/kg Physcion (dissolved in 10% DMSO and 90% saline), or (iv) 40 mg/kg NCT-503 and 20 mg/kg Physcion. Each drug or placebo was administered daily as a 200 μL intraperitoneal injection for a 14-day treatment period or until the tumour reached a maximum volume of 1000 mm^3^. Tumour volume was measured daily with a digital caliper and calculated using the following formula: V = (L × W^2^/2), where V is volume, L is length, and W is width. To determine differences in tumour growth non-liner regression was performed with the Prism program. At experiment end the tumours were harvested, weighed and snap-frozen in liquid nitrogen or fixed in 4% paraformaldehyde for histology, stained with haematoxylin and eosin, and scanned with the Aperio ImageScope. The haematoxylin ratio was determined with ImageJ.

### Data and statistical analysis

All data are presented as mean ± standard error. Data from different experimental groups were compared using one-way and two-way ANOVA and Tukey’s multiple comparison tests. For densitometry analysis, the 2 treatment groups were compared using the Mann–Whitney *t* test.

Statistical significance was defined as p < 0.05.

## Supplementary information

Supplementary Figures.
