# Supplementary material for: The effects of fructose and metabolic inhibition on hepatocellular carcinoma
Source: Sci Rep. 2020 Oct 7;10:16769. doi: 10.1038/s41598-020-73653-5 (PMC7541473; doi:10.1038/s41598-020-73653-5)
Supplement: Supplementary file 1 — Supplementary Figures. [file 41598_2020_73653_MOESM1_ESM.docx]

**The effects of fructose and metabolic inhibition on hepatocellular carcinoma**

Brittany Dewdney^1^, Mohammed Alanazy^2^, Rhys Gillman^1^, Sarah Walker^3^, Miriam Wankell^1^, Liang Qiao^2^, Jacob George^2^, Alexandra Roberts^1^, Lionel Hebbard ^1,2^

1. Department of Molecular and Cell Biology, Centre for Molecular Therapeutics, Australian Institute of Tropical Health and Medicine, James Cook University, Townsville, QLD 4811, Australia.
2. Storr Liver Centre, Westmead Institute for Medical Research, Westmead Hospital and University of Sydney, Sydney, NSW 2145, Australia.
3. Gastroenterology and Hepatology Unit, The Canberra Hospital, Woden, ACT 2606 Australia.

*Corresponding author E-mail: [lionel.hebbard@jcu.edu.au](mailto:lionel.hebbard@jcu.edu.au); phone 617 4781 5684

**Supplementary File 1**

**
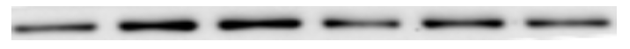
a, transketolase (TKT)** **b, transaldolase (TALDO)**

**
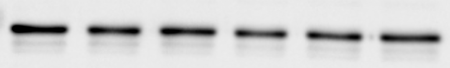
**

**68kDa -**

**Glu 24h**

**Fru 48h**

**Fru 24h**

**Fru 24h**

**Glu 48h**

**37kDa -**

**Glu 24h**

**Fru 48h**

**Glu 48h**


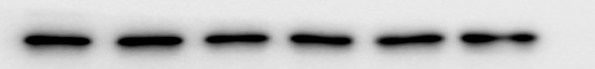

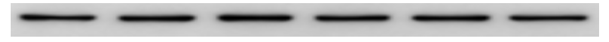


**68kDa -**

**37kDa -**

**
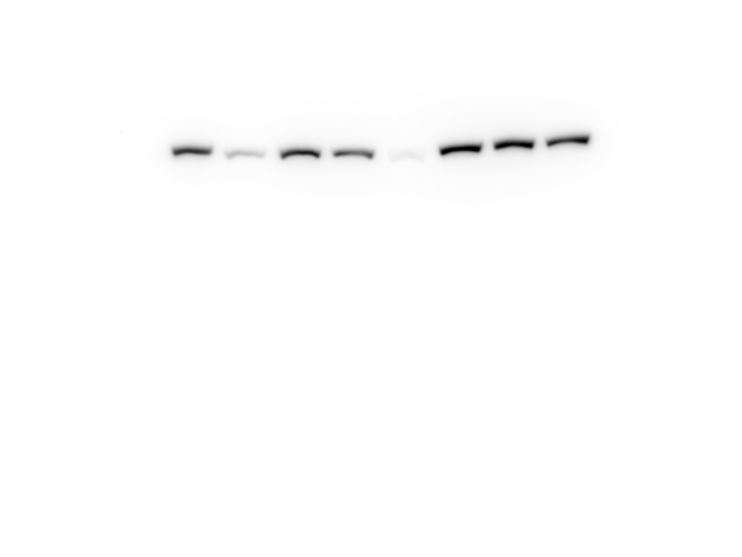

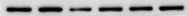

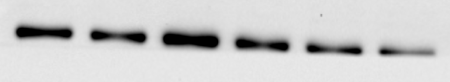
c, glucose-6-phosphate dehydrogenase (G6PD) d, phosphoglycerate dehydrogenase (PHGDH)**

**Glu 24h**

**Fru 24h**

**Glu 48h**

**Fru 48h**

**57kDa -**

**57kDa -**

**58kDa -**

**Fru 48h**

**Glu 48h**

**Fru 24h**
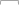


**Glu 24h**
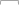


**e,** **phosphoserine aminotransferase 1 (PSAT1)** **f, β-actin**


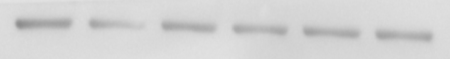


**Fru 24h**

**Glu 24h**

**Fru 24h**

**Glu 24h**

**42kDa -**

**43kDa -**

**Glu 48h**


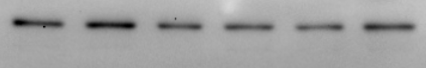
**
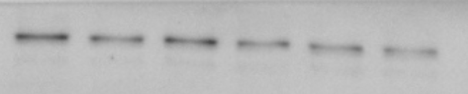
**

**Glu 48h**

**42kDa -**

**43kDa -**

**Fru 48h**

**Fru 48h**
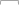


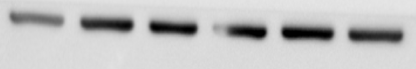


**g, β-actin**


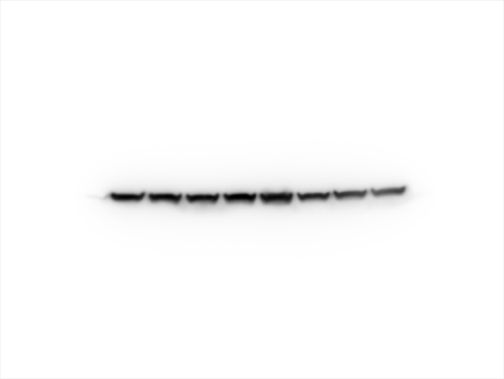


**42kDa -**

**Supplementary Figure 1. A52 HCC *in vitro* original western blots for Figure 1e.** A52 HCC cells were cultured in glucose or fructose supplemented media and protein lysates obtained after 24 and 48 hours. Protein lysates were blotted for **a**, transketolase (TKT); **b**, transaldolase (TALDO); **c**, glucose-6-phosphate dehydrogenase (G6PD); **d**, phosphoglycerate dehydrogenase (PHGDH); **e**, phosphoserine aminotransferase 1 (PSAT1); and **f**, β-actin. The labelling above indicates which samples are glucose/fructose at 24 and 48 hours and are indicated in Figure 1e of the manuscript. Images a, b, d-f were supplied from the thesis of M.A. The blot for G6PD **(c)** was performed at a different time, and the corresponding β-actin blot is shown, **g** and was performed for loading control and was used for densitometry analysis in Figure 2.

 **a**

 **b**

 **c**

 **d**

 **e**

**Supplementary Figure 2. A52 HCC *in vitro* densitometry analysis for Figure 1e.** A52 HCC *in* vitro blots from Supplementary Figure 1 were quantified and analysed using ImageJ densitometry analysis. Adjusted density value was determined by normalising to their corresponding β-actin control blot. Graphs show the mean ± standard error for **a**, transketolase (TKT); **b**, transaldolase (TALDO); **c**, glucose-6-phosphate dehydrogenase (G6PD); **d**, phosphoglycerate dehydrogenase (PHGDH); **e**, phosphoserine aminotransferase 1 (PSAT1).


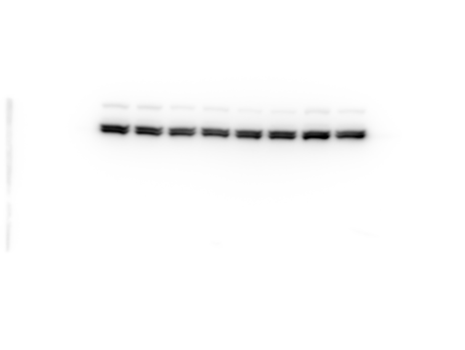

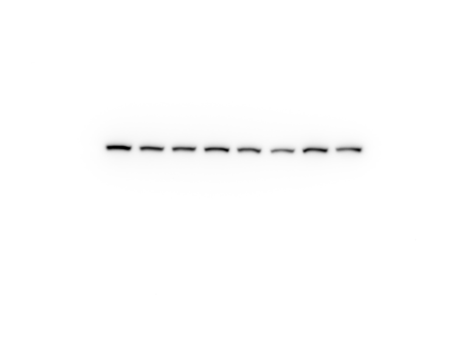
**a, transketolase (TKT)** **b, transaldolase (TALDO)**

**37kDa -**

**68kDa -**

**Fru 48h**

**Glu 48h**

**Glu 24h**
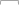


**Fru 24h**
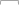


**Glu 24h**
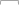


**Fru 48h**

**Glu 48h**

**Fru 24h**
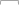


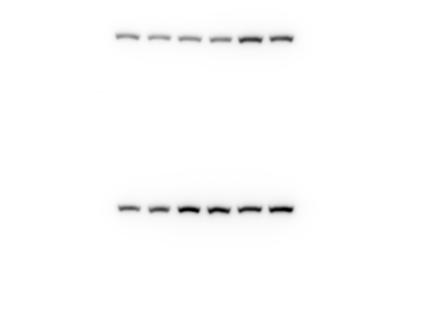
**
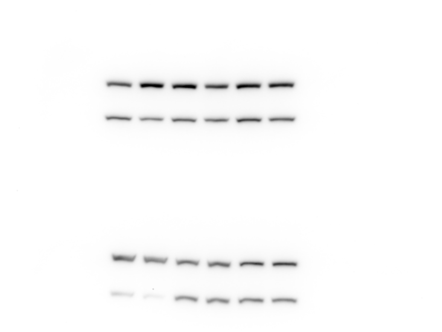
** **c, glucose-6-phosphate dehydrogenase (G6PD)**  **d, phosphoglycerate dehydrogenase (PHGDH)**

**Fru 48h**

**Glu 48h**

**Fru 24h**
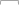


**57kDa -**

**57kDa -**

**58kDa -**

**58kDa -**

**Glu 24h**
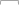


**Glu 24h**
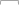


**Fru 24h**
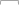


**Fru 48h**

**Glu 48h**

**
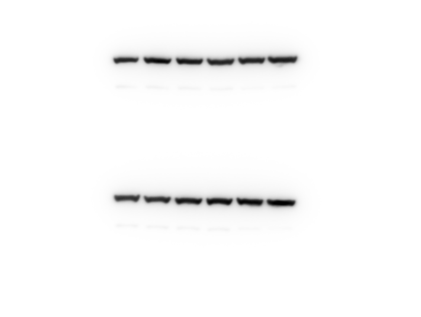

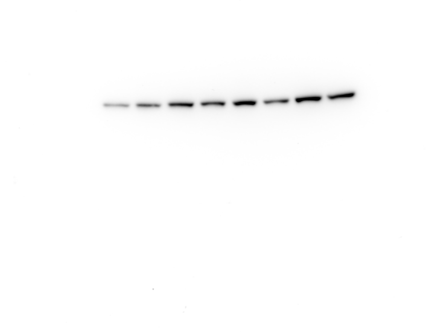
** **e,** **phosphoserine aminotransferase 1 (PSAT1)** **f, β-actin**

**42kDa -**

**42kDa -**

**43kDa -**

**Fru 48h**

**Glu 48h**

**Fru 24h**
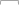


**Glu 24h**
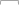


**Fru 48h**

**Glu 48h**

**Glu 24h**
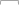


**Fru 24h**
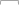


**f, β-actin**

**
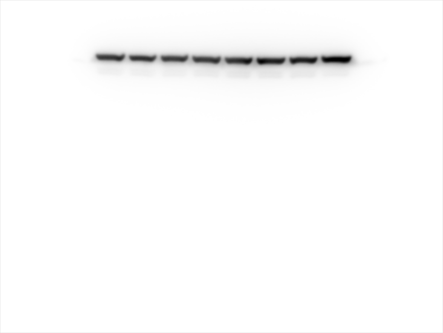
**

**42kDa -**

**­­**

**Supplementary Figure 3. Huh7 HCC *in vitro* original western blots for Figure 1f.** Huh7 HCC cells were cultured in glucose or fructose supplemented media and protein lysates obtained after 24 and 48 hours. Protein lysates were blotted for **a**, transketolase (TKT); **b,** transaldolase (TALDO); **c**, glucose-6-phosphate dehydrogenase (G6PD); **d**, phosphoglycerate dehydrogenase (PHGDH); **e**, phosphoserine aminotransferase 1 (PSAT1); and **f**, β-actin. The labelling above indicates which samples are glucose/fructose at 24 and 48 hours and are indicated in Figure 1f of the manuscript. Non-labelled bands in **c, d** and **f** represent control samples that were not included in Figure 1f of the manuscript. The blots for **a, b, and e** were performed at a different time, and the corresponding β-actin blot is shown, **g** and was performed for loading control and was used for densitometry analysis in Figure 4.

**a**

**** **b**

 **c**

 **d**

 **e**

**Supplementary Figure 4. Huh7 HCC *in vitro* densitometry analysis for Figure 1f.** Huh7 HCC *in vitro* blots from Supplementary Figure 3 were quantified and analysed using ImageJ densitometry analysis. Adjusted density value was determined by normalising to the β-actin control blot. Graphs show the mean ± standard error for **a**, transketolase (TKT); **b**, transaldolase (TALDO); **c**, glucose-6-phosphate dehydrogenase (G6PD); **d**, phosphoglycerate dehydrogenase (PHGDH); **e**, phosphoserine aminotransferase 1 (PSAT1).

**
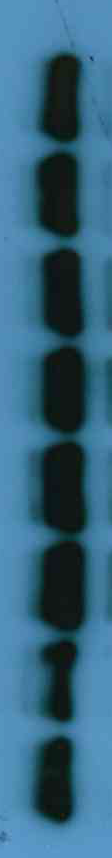
**
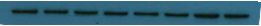
**a, transketolase (TKT)** **b, transaldolase (TALDO)**

**Normal chow**

**High fructose chow**

**High fructose chow**

**Normal chow**

**37kDa -**

**68kDa -**

**
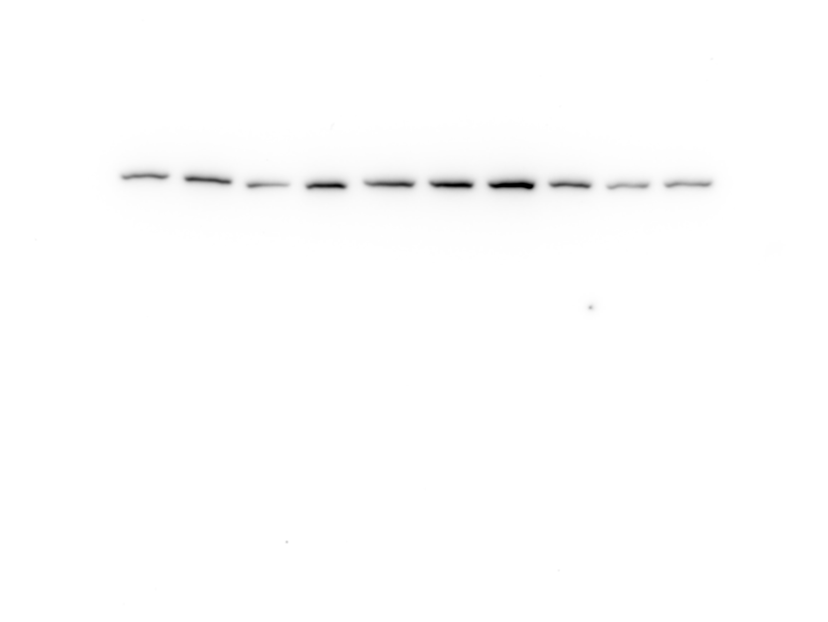

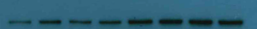
c, glucose-6-phosphate dehydrogenase (G6PD)**  **d, phosphoglycerate dehydrogenase (PHGDH)**

**57kDa -**

**58kDa -**

**Normal chow**

**High fructose chow**

**Normal chow**

**High fructose chow**


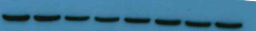
 **e,** **phosphoserine aminotransferase 1 (PSAT1)** **f, β-actin**

**Normal chow**

**High fructose chow**


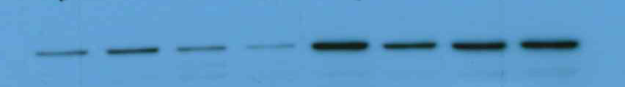


**42kDa -**

**43kDa -**

**Normal chow**

**High fructose chow**

**g, β-actin**


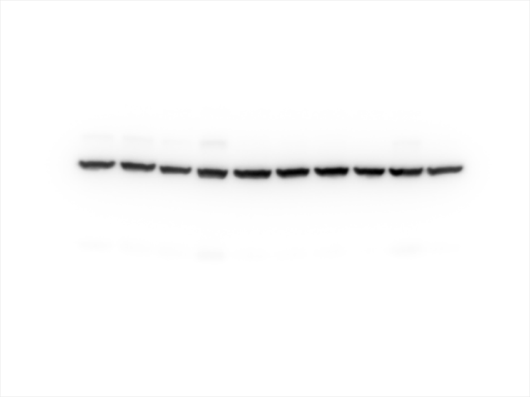


**42kDa -**

**Supplementary Figure 5. A52 HCC *in vivo* original western blots for Figure 1g.** A52 HCC tumours were harvested from normal chow and high fructose chow fed mice and protein lysates obtained. Protein lysates were blotted for **a**, transketolase (TKT); **b**, transaldolase (TALDO); **c**, glucose-6-phosphate dehydrogenase (G6PD); **d**, phosphoglycerate dehydrogenase (PHGDH); **e**, phosphoserine aminotransferase 1 (PSAT1); and **f**, β-actin. The labelling above indicates which samples are normal chow and high fructose chow. In image **c**, the original blot image was cropped to include only 4 of the 5 samples from the blot, as shown in Figure 1g in the manuscript. Images a, b, d-f were supplied from the thesis of M.A. The blot for G6PD **(c)** was performed at a different time, and the corresponding β-actin blot is shown, **g,** and was performed for loading control and was used for respective densitometry analysis in Figure 6.

 **a b**

** c d**

 **e**

**Supplementary Figure 6. A52 HCC *in vivo* densitometry analysis for Figure 1g.** A52 HCC tumour *in vivo* blots from Supplementary Figure 5 were quantified and analysed using ImageJ densitometry analysis. Adjusted density value was determined by normalising to the B-actin control blot. Graphs show the mean ± standard error for **a**, transketolase (TKT); **b**, transaldolase (TALDO); **c**, glucose-6-phosphate dehydrogenase (G6PD); **d**, phosphoglycerate dehydrogenase (PHGDH); **e**, phosphoserine aminotransferase 1 (PSAT1). * p<0.05

**
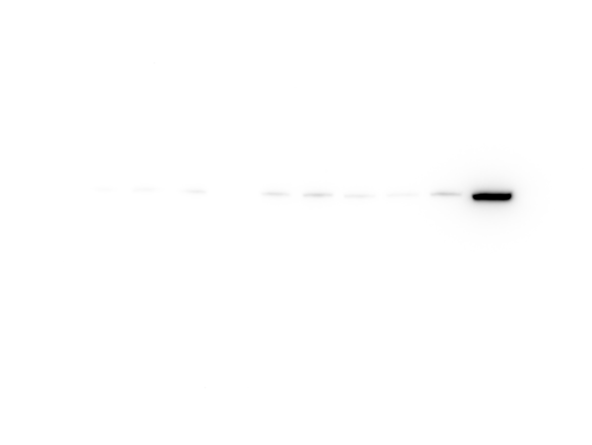

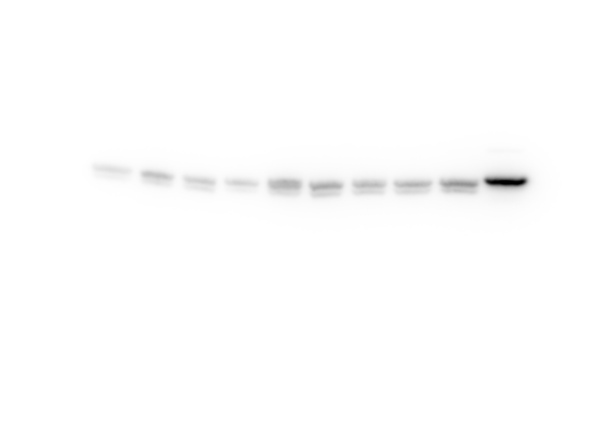
 a, transketolase (TKT)**  **b, transaldolase (TALDO)**

**37kDa -**

**68kDa -**

**High fructose chow**

**Normal chow**

**Normal chow**

**High fructose chow**

**
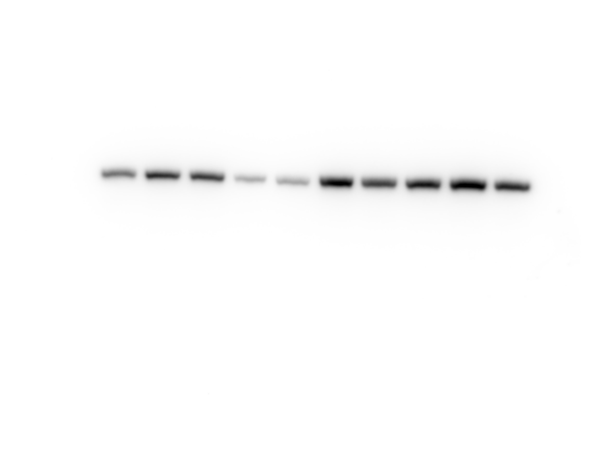
**
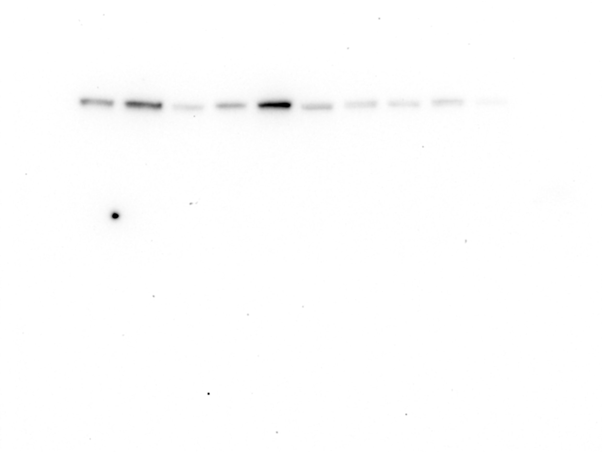
**c, glucose-6-phosphate dehydrogenase (G6PD)**  **d, phosphoglycerate dehydrogenase (PHGDH)**

**57kDa -**

**58kDa -**

**Normal chow**

**High fructose chow**

**Normal chow**

**High fructose chow**

**
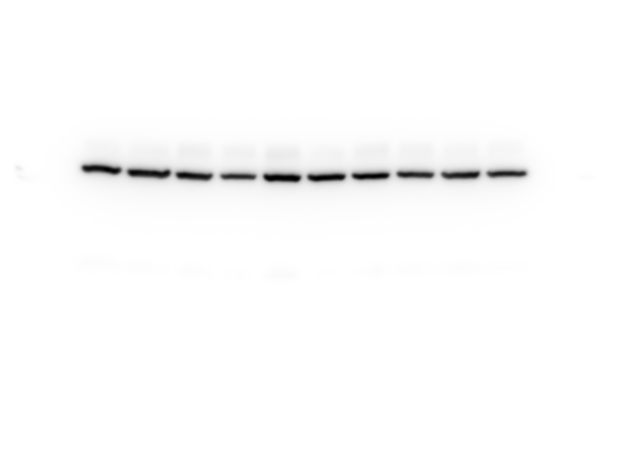

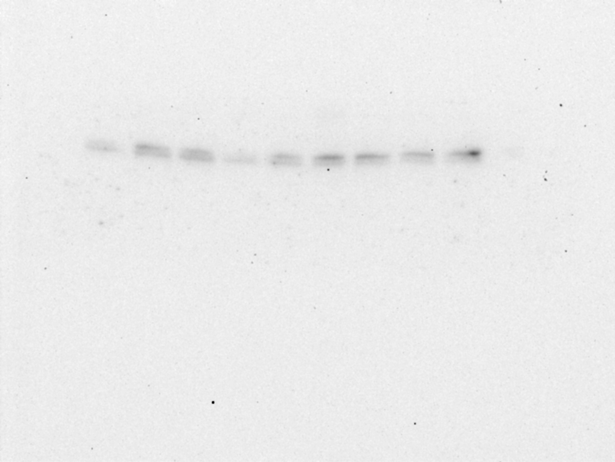
 e,** **phosphoserine aminotransferase 1 (PSAT1)** **f, β-actin**

**42kDa -**

**43kDa -**

**Normal chow**

**High fructose chow**

**High fructose chow**

**Normal chow**

**Supplementary Figure 7. Huh7 HCC *in vivo* original western blots for Figure 1h.** Huh7 HCC tumours were harvested from normal chow and high fructose chow fed mice and protein lysates obtained. Protein lysates were blotted for **a**, transketolase (TKT); **b**, transaldolase (TALDO); **c**, glucose-6-phosphate dehydrogenase (G6PD); **d**, phosphoglycerate dehydrogenase (PHGDH); **e**, phosphoserine aminotransferase 1 (PSAT1); and **f**, β-actin. The labelling above indicates which samples are normal chow and high fructose chow. In all images**,** the original blot image was cropped to include only 4 of the 5 samples from the blot, as shown in Figure 1h in the manuscript

 **a b**


 **c d**

** e**

**Supplementary Figure 8. Huh7 HCC *in vivo* densitometry analysis for Figure 1h.** Huh7 HCC tumour *in vivo* blots from Supplementary Figure 6 were quantified and analysed using ImageJ densitometry analysis. Adjusted density value was determined by normalising to the B-actin control blot. Graphs show the mean ± standard error for **a**, transketolase (TKT); **b**, transaldolase (TALDO); **c**, glucose-6-phosphate dehydrogenase (G6PD); **d**, phosphoglycerate dehydrogenase (PHGDH); **e**, phosphoserine aminotransferase 1 (PSAT1). * p<0.05; ** p<0.01

**Supplementary Figure 9. A52 and Huh7 tumours haematoxylin ratios.** A52 and Huh7 tumours were stained with haematoxylin and eosin and the haematoxylin percentage was quantified using Fiji. A52 normal chow placebo, n=6; A52 normal chow NCT-503, n=8; A52 normal chow Physcion, n=8; A52 normal chow NCT-503 + Physcion, n=10; A52 fructose chow placebo, n=8; A52 fructose chow NCT-503, n=6; A52 fructose chow Physcion, n=8; A52 fructose chow NCT-503 + Physcion, n=9; Huh7 normal chow placebo, n=7; Huh7 normal chow NCT-503, n=8; Huh7 normal chow Physcion, n=7; Huh7 normal chow NCT-503 + Physcion, n=11; Huh fructose chow placebo, n=5; Huh7 fructose chow NCT-503, n=6; Huh7 fructose chow Physcion, n=6; Huh7 fructose chow NCT-503 + Physcion, n=7.


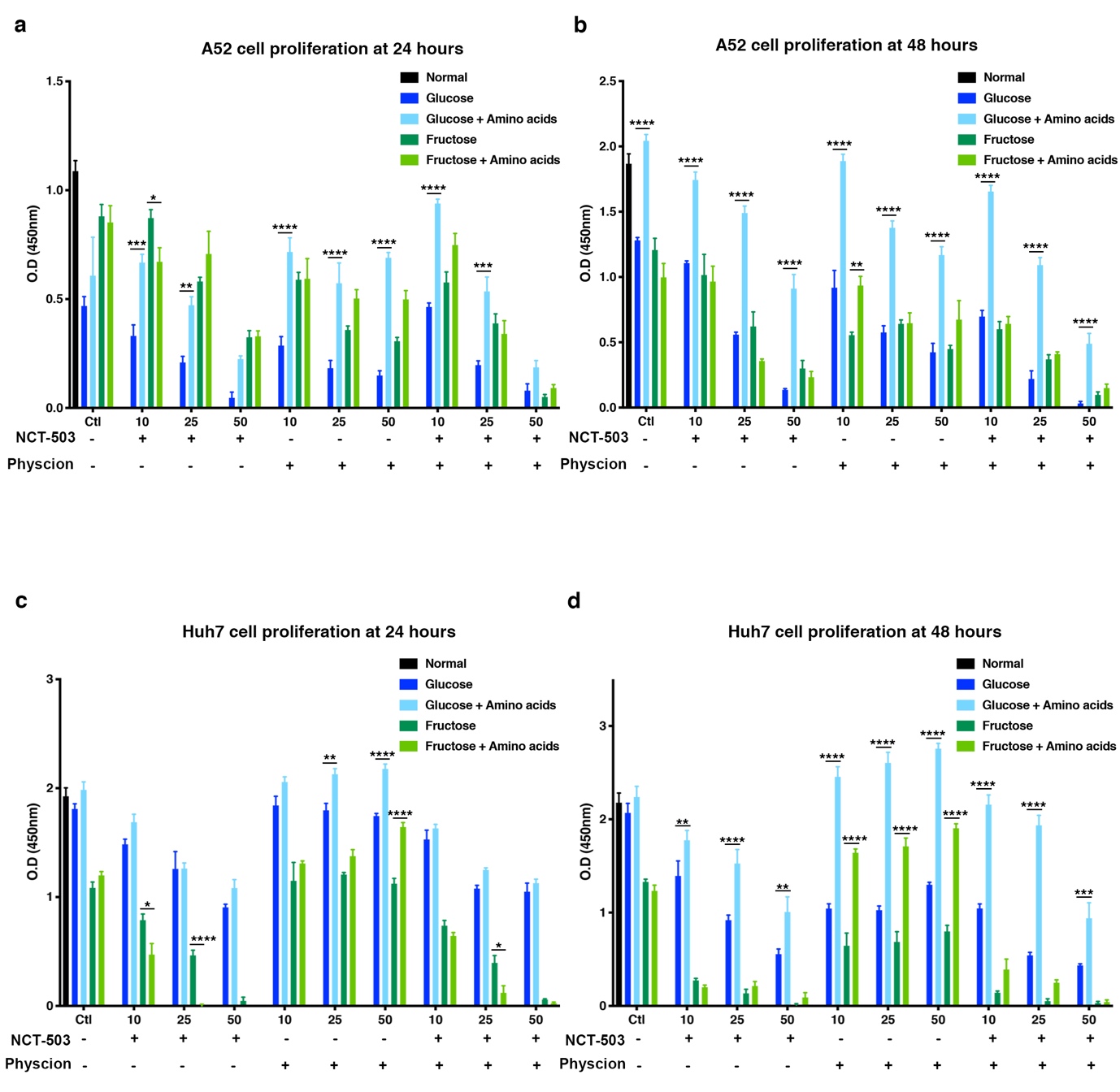


**Supplementary Figure 10. A52 and Huh7 cell proliferation with NCT-503, Physcion, and amino acids.** Cell proliferation was determined using the BrdU assay in cells grown in either normal media, media containing 5 mM glucose or 5 mM fructose, with or without the addition of non-essential amino acids, and with or without 10, 25, or 50 uM of NCT-503 and/or Physcion. Graphs represent the average optical density at 450 nm (n=4) for **a**, A52 cells at 24 hours; **b**, A52 cells at 48 hours; **c**, Huh7 cells at 24 hours; and **d**, Huh7 cells at 48 hours, after drug treatments. * p<0.05; ** p<0.01; *** p<0.001; **** p<0.0001. Ctl, control.
